# The spatial transmission of SARS-CoV-2 in China under the prevention and control measures at the early outbreak

**DOI:** 10.1186/s13690-021-00529-z

**Published:** 2021-01-13

**Authors:** Jianli Liu, Yuan Zhou, Chuanyu Ye, Guangming Zhang, Feng Zhang, Chunjuan Song

**Affiliations:** 1 School of Textile Science and Technology, Jiangnan University, Wuxi, 214122 China; 2 The Second Affiliated Hospital of Soochow University, Suzhou, 215123 China; 3 The University of Texas Health Science Center at Houston, TX77030, Houston, USA; 4 School of Biology and Basic Medical Sciences, Soochow University, Suzhou, 215123 China; 5 The First Affiliated Hospital of Nanjing Medical University, Nanjing, 210029 China

**Keywords:** COVID-19, Early outbreak phase, Generalized-growth model, Prevention and control measures, Spatial transmission, Basic reproduction number

## Abstract

**Background:**

Since severe acute respiratory syndrome coronavirus, 2 (SARS-CoV-2) was firstly reported in Wuhan City, China in December 2019, Novel Coronavirus Disease 2019 (COVID-19) that is caused by SARS-CoV-2 is predominantly spread from person-to-person on worldwide scales. Now, COVID-19 is a non-traditional and major public health issue the world is facing, and the outbreak is a global pandemic. The strict prevention and control measures have mitigated the spread of SARS-CoV-2 and shown positive changes with important progress in China. But prevention and control tasks remain arduous for the world. The objective of this study is to discuss the difference of spatial transmission characteristics of COVID-19 in China at the early outbreak stage with resolute efforts. Simultaneously, the COVID-19 trend of China at the early time was described from the statistical perspective using a mathematical model to evaluate the effectiveness of the prevention and control measures.

**Methods:**

In this study, the accumulated number of confirmed cases publicly reported by the National Health Committee of the People’s Republic of China (CNHC) from January 20 to February 11, 2020, were grouped into three partly overlapping regions: Chinese mainland including Hubei province, Hubei province alone, and the other 30 provincial-level regions on Chinese mainland excluding Hubei province, respectively. A generalized-growth model (GGM) was used to estimate the basic reproduction number to evaluate the transmissibility in different spatial locations. The prevention and control of COVID-19 in the early stage were analyzed based on the number of new cases of confirmed infections daily reported.

**Results:**

Results indicated that the accumulated number of confirmed cases reported from January 20 to February 11, 2020, is well described by the GGM model with a larger correlation coefficient than 0.99. When the accumulated number of confirmed cases is well fitted by an exponential function, the basic reproduction number of COVID-19 of the 31 provincial-level regions on the Chinese mainland, Hubei province, and the other 30 provincial-level regions on the Chinese mainland excluding Hubei province, is 2.68, 6.46 and 2.18, respectively. The consecutive decline of the new confirmed cases indicated that the prevention and control measures taken by the Chinese government have contained the spread of SARS-CoV-2 in a short period.

**Conclusions:**

The estimated basic reproduction number thorough GGM model can reflect the spatial difference of SARS-CoV-2 transmission in China at the early stage. The strict prevention and control measures of SARS-CoV-2 taken at the early outbreak can effectively reduce the new confirmed cases outside Hubei and have mitigated the spread and yielded positive results since February 2, 2020. The research results indicated that the outbreak of COVID-19 in China was sustaining localized at the early outbreak stage and has been gradually curbed by China’s resolute efforts.

## Background

The Covid-19 has been the most extensive global pandemic to afflict humanity in a century [1]. Facing this unknown, unexpected, and devastating disease, prevention, and control of COVID-19 are a great challenge for the entire world. On December 27, 2019, cases of pneumonia of unknown cause were first identified in Wuhan, the capital city of Hubei province and the biggest city in China’s central region [2]. On January 19, 2020, the spreading of COVID-19 between humans was determined [3]. During this period, community spread and clusters of cases emerged in Wuhan, and confirmed cases were also reported in other Chinese regions, which were due to virus carriers traveling before the most important traditional holiday, the Spring Festival, in China [4]. Since January 20, 2020, the spread of COVID-19 in the Chinese mainland became most pressing with the rapid increase in new confirmed cases. As a crucial step to contain the spread of SARS-CoV-2, the Chinese government took the decisive measure to close outbound traffic from Wuhan city and Hubei province on January 23, 2020. Hubei province was the epicenter of the COVID-19 epidemic in China. Since December 31, 2019, and as of November 30, 2020, more than sixty-two million cases of COVID-19 have been officially reported by the World Health Organization in 216 countries, areas, or territories, and is likely to claim many more [5].

COVID-19 research has already caught the attention of the majority of scholarly communications communities in 2020. The analysis and modeling of COVID-19 data have been one of the research topics. Computational simulation and modeling of the existed COVID-19 data have played an important role to predict the development of the current pandemic [6]. The well-known SEIR (Susceptible-Exposed-Infectious-Recovered) and modified SEIRS models have been widely used to model epidemics including severe acute respiratory syndrome **(**SARS), middle east respiratory syndrome (MERS), and COVID-19 [7, 8]. Singhal, Singh, Lall, et al. used a Gaussian mixture model to model and predicted the COVID-19 pandemic in India, the prediction results agree well with a very popular study based on the classic susceptible-infected-recovered (SIR) model [9]. Ayinde, Lukman, Rauf, et al., used statistical models in simple, quadratic, cubic, and quartic forms to model Nigerian COVID-19 cases, and found out that daily cumulative forecast values of the Least Absolute Deviation estimator for May and June 2020 with a 99% confidence level [10]. By considering the effects of prevention and control measures and the increase of the public’s prevention awareness, Zheng, Du, and Wang, et al. embedded the natural language processing module and the artificial neural network into the improved susceptible-infected (ISI) model to build the hybrid artificial intelligence (AI) model for COVID-19 prediction in China [11]. Gao, Zhang, and Yao, et al. proposed using a Boltzmann function-based model to forecast the cumulative number of COVID-19 death in China with high confidence [12]. In theory, the prediction of the trend of COVID-19 is a special case of pattern recognition, which provides a tool to unravel the hidden rules and principles of an unknown and novel epidemic from the perspective of mathematics. Models can help the governments to make decisive policies to contain the spread of SARS-CoV-2. But, we should distinguish between what the models can and cannot predict. However, we also should realize that all proposed models are incomplete to describe the real-world system, especially for an epidemic or pandemic period [13].

To analyze the difference of spatial transmission characteristics of COVID-19 in China at the early outbreak stage with the joint epidemic prevention and control mechanism was activated across China, the accumulated number of confirmed cases publicly reported by China’s National Health Commission (NHC) from January 20 to February 11, 2020, were clustered into three groups according to the spatial locations, e.g., 31 provincial-level regions on the Chinese mainland, Hubei province, and the other 30 provincial-level regions on the Chinese mainland excluding Hubei province, respectively. Then, the preliminary estimation of an epidemic mathematical model to characterize the early rapid ascending phase outbreak at different spatial locations was carried out, and the basic reproduction number *R*_0_ was estimated when the fitted GGM of the accumulated number of confirmed cases exhibits global exponential growth.

## Methods

The objective of this study is to discuss the difference in spatial transmission characteristics of COVID-19 and analyze the effectiveness of the prevention and control measures of COVID-19 in China at the early outbreak stage. To characterize the transmission properties of an epidemic at the early outbreak stage mathematical models have been widely used [14]. Modeling enables early indications on the future projections of the pandemic and is useful to estimate the efficiency of control actions in the battle against the COVID-19. A data-driven epidemic prediction model could provide a quantitative framework to explain the reported data, improve the epidemic scale forecast, assess and optimize the impact of interventions and control strategies through data mining from the perspective of statistical learning theory [15]. The generalized-growth model (GGM) is a typical data-driven model, which only uses the publicly reported data without a complex hypothesis and prior knowledge. GGM is one of the useful models to characterize and forecast early outbreak at the ascending phase of the epidemic. GGM is described by the following differential equations,

1 $$ {C}^{\prime }(t)={rC}^p(t) $$

2 $$ {C}^{\prime }(t)=\frac{dC(t)}{dt} $$

Where *C*^′^(*t*) describes the confirmed cases growth rate of *C*(*t*) at time *t*, *C*(*t*) represents the accumulated number of confirmed cases at time *t*. If the daily reported new cases of confirmed infections are considered as *C*^′^(*t*) the interval of the time series *C*(*t*) is 1 day. *r* and *p* are two important parameters that control the growth rate and the deceleration of growth of *C*(*t*) [15]. The accumulated number of confirmed cases in the Chinese mainland, the other 30 provincial-level regions on the Chinese mainland excluding Hubei province, and Hubei Province was firstly reported in multiple forms by CNHC on January 20, 2020. In this study, the accumulated number of confirmed cases publicly reported by CNHC was used as *C*(*t*) from January 20 to February 11, 2020. We denote the daily accumulated number of confirmed cases of Hubei province and other 30 provincial-level regions on the Chinese mainland excluding Hubei province, and the 31 provincial-level regions Chinese mainland including Hubei province by *C*_*h*_(*t*), *C*_*o*_(*t*) and *C*_*m*_(*t*), respectively. Here, *t* = 1⋯23 is a time series of the outbreak of COVID-19 from January 20 to February 11, 2020.

When the GGM is built using *C*_*h*_(*t*), *C*_*o*_(*t*) or *C*_*m*_(*t*), the value of *p* that determines the type of GGM, which varies between 0 to 1 [16]. If *p* = 0, GGM is a linear function. While *p* = 1 GGM is an exponential function. If 1 > *p* > 0, then GGM is a polynomial function (e.g. polynomial). The basic reproductive number *R*_0_ is defined as the expected number of secondary infectious cases generated by an average infectious case in an entirely susceptible population [17]. *R*_0_ is not only used to describe the transmission characteristics but evaluate the effectiveness of intervention measures. If the number of new cases of confirmed infections daily reported following an exponential distribution at the early outbreak phase, the corresponding mathematical model is as the following,

3 $$ {C}^{\prime }(t)=C(t){e}^{\left(\beta -\gamma \right)t} $$

4 $$ {R}_0=\frac{\beta }{\gamma } $$

5 $$ {C}^{\prime }(t)=C(t)-C\left(t-1\right) $$

where *C*^′^(*t*) is the number of new cases of confirmed infections at time *t*, *β* is the mean transmission rate, and *γ* is the reciprocal of mean infectious period [16]. The value of *β* and *γ* will be calculated for the estimation of *R*_0_ using the algorithm proposed by Chowell [15, 18].

## Results and discussion

### Data preparation and analysis

A daily briefing on COVID-19 in 31 provincial-level regions on the Chinese mainland was officially published by CNHC from January 20, 2020. We used data from January 20 to February 11, 2020, 2020, based on the accumulated number of confirmed cases publicly reported by CNHC. The data are listed in Table 1. We denote the daily accumulated number of confirmed cases of Hubei province and other 30 provincial-level regions on the Chinese mainland excluding Hubei province, and the 31 provincial-level regions of Chinese mainland including Hubei province by *C*_*h*_(*t*), *C*_*o*_(*t*) and *C*_*m*_(*t*), respectively. Here, *t* = 1⋯23 is a time series of the outbreak of COVID-19 from January 20 to February 11, 2020.

**Table 1:** Data provided by CNHC from January 20 to February 11, 2020

Date	*t* (days)	*C*_*m*_(*t*)	*C*_*h*_(*t*)	*C*_*o*_(*t*)
January 20	1	291	270	21
January 21	2	440	375	65
January 22	3	571	444	127
January 23	4	830	549	281
January 24	5	1287	729	558
January 25	6	1975	1052	923
January 26	7	2744	1423	1321
January 27	8	4515	2714	1801
January 28	9	5974	3554	2420
January 29	10	7711	4586	3125
January 30	11	9692	5806	3886
January 31	12	11,791	7153	4638
February 1	13	14,380	9074	5306
February 2	14	17,205	11,177	6028
February 3	15	20,438	13,522	6916
February 4	16	24,324	16,678	7646
February 5	17	28,018	19,665	8353
February 6	18	31,161	22,112	9049
February 7	19	34,546	24,953	9593
February 8	20	37,198	27,100	10,098
February 9	21	40,171	29,631	10,540
February 10	22	42,638	31,728	10,910
February 11	23	44,653	33,366	11,287

In this study, we first built three mathematical models *f*_*h*_(*t*), *f*_*o*_(*t*) and *f*_*m*_(*t*), using time series data shown in Table 1. The built model typically varies according to the time scale when the GGM algorithm is used. After the model building, we calculated the basic reproduction number *R*_0_ with eq. 4 when the built model exhibits global exponential growth rates. Each *R*_0_ will be calculated for each built model, which will constitute a time series ${R}_0^l(t)$, and *l* = *h*, *o* or *m*, has the same meaning as mentioned above.

**Fig. 1:**
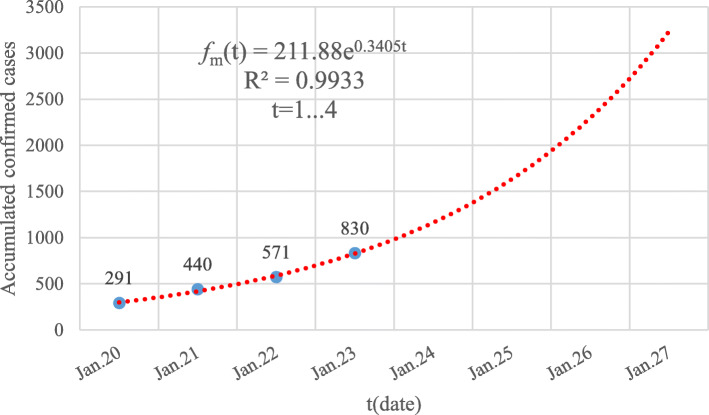
The GGM model of the accumulated number of confirmed cases of the Chinese mainland from January 20 to January 23, 2020. The blue dots are the reported data while the dotted red line corresponds to the built GGM model

To ensure the robustness and prediction precision of the built model using the GGM algorithm, at least 4 observed data are used for modeling [19]. So, the construction of *f*_*h*_(*t*), *f*_*o*_(*t*) and *f*_*m*_(*t*) are based on the accumulated number of confirmed cases from January 20 January 23, 2020. The best fit of the GGM model of *f*_*m*_(*t*) is shown in Fig. 1.

**Fig. 2:**
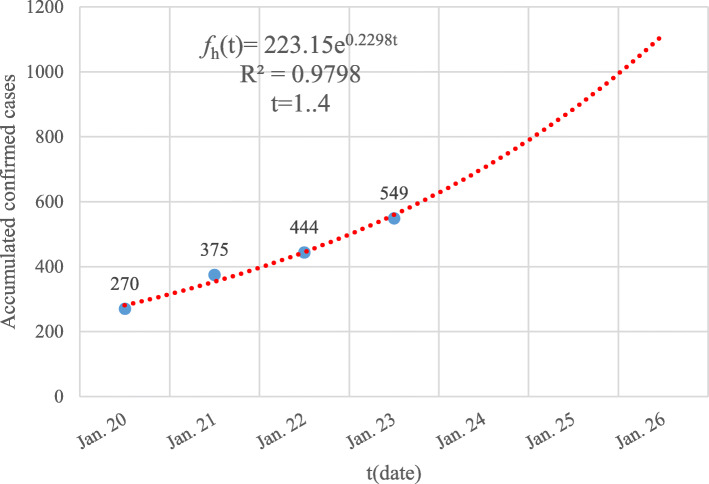
The GGM model of the accumulated number of confirmed cases of Hubei province from January 20 to January 23, 2020. The blue dots are the reported data while the dotted red line corresponds to the built GGM model

**Fig. 3:**
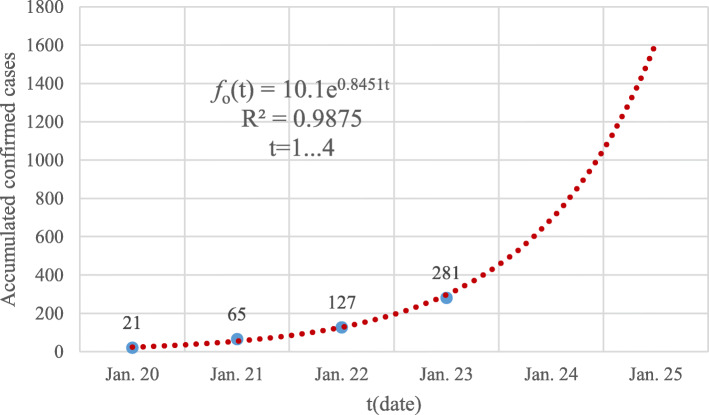
The GGM model of the accumulated number of confirmed cases of the other 30 provincial-level regions on the Chinese mainland excluding Hubei province from January 20 to January 23, 2020. The blue dots are the reported data while the dotted red line corresponds to the built GGM model

Similarly, the GGM models of *f*_*h*_(*t*) and *f*_*o*_(*t*) were also fitted like the ones illustrated in Fig. 2 and Fig. 3, respectively.

**Fig. 4:**
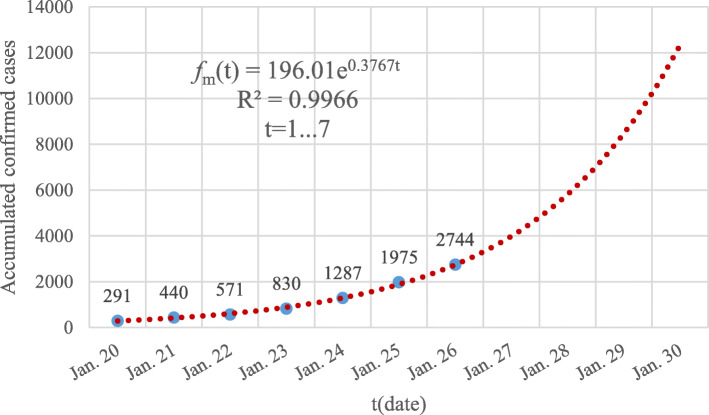
The GGM model of the accumulated number of confirmed cases of the Chinese mainland from January 20 to January 26, 2020. The blue dots are the reported data while the dotted red line corresponds to the built GGM model

The four-day ahead GGM models of *f*_*h*_(*t*), *f*_*o*_(*t*) and *f*_*m*_(*t*) are all exponential functions. We also found that the seven-day ahead GGM model of *f*_*m*_(*t*), the six-day ahead GGM model of *f*_*h*_(*t*) and the five-day ahead GGM model of *f*_*o*_(*t*) are all exponential functions with a higher correlation coefficient *R*^2^ ≥ 0.99. The GGM model of the first 7 days of the accumulated number of confirmed cases of the Chinese mainland is shown in Fig. 4.

The seven-day ahead GGM model of the accumulated number of confirmed cases of a Chinese mainland sharply increases when compared with the four-day ahead GGM model because of the obvious differences among the model parameters, *r* and *p*. A similar conclusion also can be induced from the GGM models of *f*_*h*_(*t*) and *f*_*o*_(*t*) when the observing time scale *t* varies from a shorter one to a longer one.

**Fig. 5:**
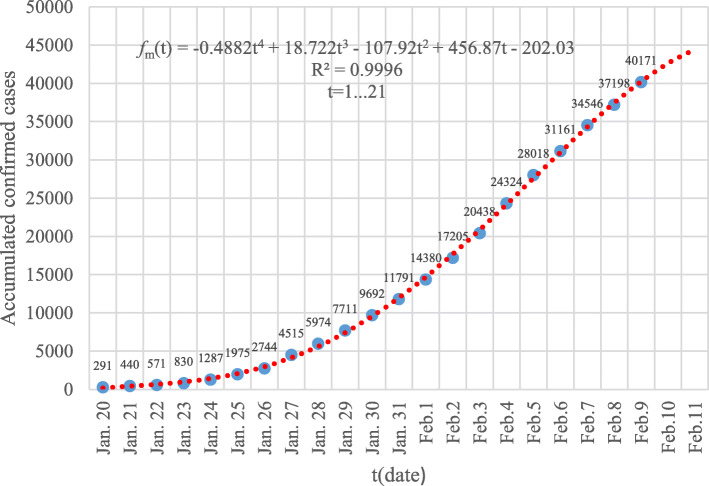
The GGM model of the accumulated number of confirmed cases of the Chinese mainland from January 20 to February 11, 2020. The blue dots are the reported data while the dotted red line corresponds to the built GGM model

When the observed time scale *t* of COVID-19 is extended, the GGM model of *f*_*h*_(*t* ≥ 7), *f*_*o*_(*t* ≥ 6) and *f*_*m*_(*t* ≥ 8) will be best fitted with sub-exponential (i.e. polynomial) function. As shown in Fig. 5, the GGM model of *f*_*m*_(*t*) based on the accumulated number of confirmed cases from January 20 to February 9, 2020, is well fitted with a polynomial of degree four. The correlation coefficient *R*^2^ between the reported data and the fitted ones is larger than 0.99. Similarly, the sub-exponential growth dynamic is a common phenomenon of epidemics of COVID-19 when the accumulated number of confirmed cases in Hubei Province and the other 30 provincial-level regions on the Chinese mainland excluding Hubei province are evaluated through GGM model. The early outbreak of COVID-19 exhibits both global polynomial and local exponential growth pattern.

### Estimation of basic reproduction number using GGM model

We estimated the basic reproduction number *R*_0_ with eq. 4 for three different GGM models when they exhibit global exponential growth rates.

${R}_0^m(7)=2.68$(95% CrI 2.19–3.12), the corresponding GGM model of *f*_*m*_(*t*) is *f*_*m*_(*t*) = 196.01*e*^0.3767*t*^, *t* = 1⋯7.

${R}_0^h(6)=6.46$ (95% CrI 4.38–9.17), the corresponding GGM model of *f*_*h*_(*t*) is *f*_*h*_(*t*) = 209.75*e*^0.2573*t*^, *t* = 1⋯6.

${R}_0^o(5)=2.18$ (95% CrI 1.61–3.09), the corresponding GGM model of *f*_*o*_(*t*) is *f*_*o*_(*t*) = 11.002*e*^0.8024*t*^, *t* = 1⋯5.

The basic reproduction number *R*_0_ for each GGM model was computed along with a 95% confidence interval. The two built models *f*_*h*_(*t*) and *f*_*o*_(*t*), represent the transmission at the early outbreak phase of 2019-nCoV in two different spatial locations. The basic reproduction number of the 31 provincial-level regions Chinese mainland including Hubei province is 2.68, which is coincident with the estimated result in Wu’s work [8].

In the research of Wu and his collaborators, the basic reproduction number was 2.68 (95% CrI 2.47–2.86) estimated by SEIR model by using the data from December 31, 2019, to January 28, 2020, in Wuhan city, China [8]. It’s that more than 90% of the confirmed cases at the early stage were in Wuhan city, the original epicenter of China’s COVID-19 outbreak [20]. Wang, et al. employed the Markov Chain Monte Carlo algorithm to estimate the basic reproduction number that is 5.78 (95% CrI 5.71–5.89) by using the number of cumulative confirmed cases from 10 January to 4 February 2020 in mainland China [21]. It is worth noting that February 4 is an important turning point in the COVID-19 in China, and the transmission trend was under control [22]. This is the main reason responsible for the relatively large basic reproduction number when compared with our result. Wan, et al. proposed a COVID-19 transmission dynamic model to estimate the transmission risk of COVID-19 in the mainland of China excluding Hubei province by using the data including the cumulative confirmed cases, the cumulative deaths, newly confirmed cases, and the cumulative recovered cases between 20 January and 3 March 2020 [23]. The calculated basic reproduction number is 3.36 (95% CrI 3.20–3.64) in reference [23], which is a description of a prolonged transmission period under the strict containment strategies implemented by the Chinese government. According to the systematic review of the modeling of transmission dynamics of COVID-19 by Guan, Wei, and Yang, the basic reproduction numbers at different stages are affected by the control measures and are not constant [6].

### The difference of spatial transmission of SARS-CoV-2 at the early outbreak

A higher *R*_0_ of *f*_*h*_(*t*) for Hubei province was estimated because of the capital of Hubei province, Wuhan city, which is the epicenter of the COVID-19 outbreak. No COVID-19-specific nationwide interventions were carried out before January 23, 2020, because of the great uncertainty about the new disease. More research was needed to understand its mode of transmission and the risk of human-to-human transmissibility. The spread model of SARS-CoV-2 is very complicated. There is no doubt that the massive migration and gathering of people during the Chinese Lunar New Year accelerated the rapid transmission in the first several weeks of the outbreak. The severe transmission of asymptomatic carriers was not taken into consideration at the early stage because of the shortage of test kits, which aided the transmission rate of COVID-19 in Hubei Province at the early stage.

A lower *R*_0_ of *f*_*o*_(*t*) for the model of the other 30 provincial-level regions on the Chinese mainland excluding Hubei province was estimated accounting for the outbreak of COVID-19 in these regions are caused by some imported cases and the effect of the substantial public health interventions. More effective epidemic prevention and control measures were carried out to reduce the risk of COVID-19 transmission through passenger transportation. On January 22, 2020, the immediate imposition of tight restrictions on the movement of people and channels of exit in Hubei and Wuhan was carried out. At the same time, the Ministry of Transport issued an emergency circular suspending passenger traffic into Wuhan from other parts of the country by road or waterway. On January 23, 2020, the CNHC and five other government departments also issued Notice on Preventing the Transmission of Novel Coronavirus Pneumonia via Means of Transport. From January 23 to January 29, 2020, all provinces and equivalent administrative units on the Chinese mainland activated Level 1 public health emergency response. These resolute efforts and rigorous measures are effective to stem the spread of SARS-CoV-2 within Hubei province and beyond at the early outbreak stage.

The differences between the estimated *R*_0_ also indicate that COVID-19 are sustaining localized outbreaks in some neighboring provinces and the ones with related industry chain and close economic partnership. While the transmissibility of COVID-19 is not similar everywhere domestically over time because of the imported infectious cases from Hubei province have been effectively controlled through the metropolitan-wide and rural quarantine in several provinces. Some rural areas in the neighboring provinces, such as Hunan province, Henan province, Anhui province, Jiangxi province and Chongqing city (municipality directly under the Central Government of China) with close transport have also become potential outbreak epicenters because of the imported cases (e.g., migrant workers working in Hubei province). Although Zhejiang province and Guangdong province are not contiguous to Hubei province, the related industry chain and close economic partnership are two important reasons responsible for the imported cases during the Spring Festival.

### Achievements of the prevention and control measures at the early outbreak

Although COVID-19 has no longer been circulated in Hubei province since January 2020, the potential of sustaining localized outbreaks out of Hubei province has been eliminated because of great efforts to prevent and control COVID-19 at all levels in China. Extraordinary measures have been taken during mass population movements at Lunar New Year since January 23, 2020, including nationwide quarantine, access restrictions of urban communities and rural villages, close of school and business, suspension of flights and trains into and out of Wuhan, suspension of public transportation, service, and entertainment industries, extended holiday of the Spring Festival, even cash rewards for informing on people who came from Hubei province [24].

**Fig. 6:**
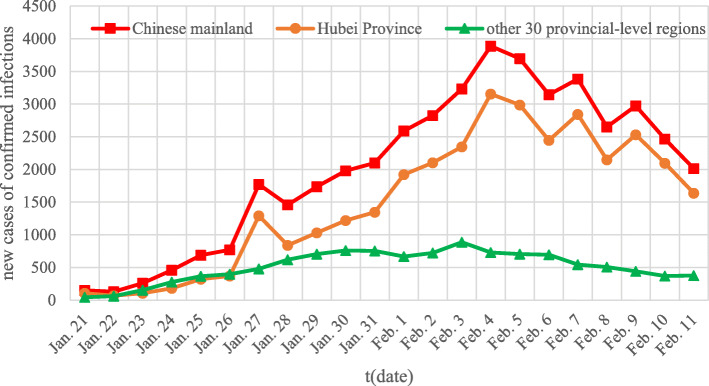
The curve of the number of new cases of confirmed infections

As shown in Fig. 6, new confirmed cases of the novel coronavirus pneumonia have seen their seventh consecutive day of decline in the Chinese mainland outside Hubei Province from February 3, 2020 (e.g., t = 15 in Fig. 6). The successive decrease of new confirmed cases indicates the strong prevention and control measures have mitigated the spread and yielded positive results. The epidemic trend of COVID-19 can also be forecasted by the daily reported number of new cases of confirmed infections, as shown in Fig. 6.

At the early outbreak phase, the number of new cases of confirmed infections sharply increases in both Hubei province and the other 30 province-level regions of the Chinese mainland excluding Hubei province. The number of new cases of confirmed infections of Hubei province accounts for the majority because of the effect of the metropolitan-wide quarantine of Wuhan and surrounding cities since January 23, 2020. At the same time, Hubei province was not only improving its ribonucleic acid (RNA) testing capability but also adding more medical workers from other provinces to diagnose the suspected cases as soon as possible to control possible transmission and offer the patients proper treatment. Since February 2, 2020, Wuhan has adopted measures to put the confirmed cases, suspected cases, febrile patients who might be carriers, and close contacts under classified management in designated facilities. The policy of ensuring that all those in need are tested, isolated, hospitalized, or treated was implemented. Actions were taken to conduct mass screenings to identify people with infections, hospitalize them, and collect accurate data on case numbers.

As shown in Fig. 6, the trend of the new cases of confirmed infections daily reported in Hubei province is also stepping into decrease phase with small swing due to many patients are unable to get treated promptly because of strain on medical resources there. Since February 2, 2020, a total of 346 medical teams composed of 42,600 medical workers and 965 public health workers from across the country and the armed forces were dispatched to support Wuhan city in fighting the epidemic. Nineteen provincial-level regions on the Chinese mainland have paired up with 16 cities across Hubei to provide medical aid with a massive influx of equipment and supplies since February 10, 2020. Two makeshift hospitals (SARS treatment-model) for the treatment of infected patients, Huoshenshan Hospital and Leishenshan Hospital built in 10 days with a total of 2900 beds have started receiving and treating patients from February 2 and February 8, 2020, respectively. The number of designated hospitals in Wuhan has risen to 45, and 16 temporary hospitals, which were converted from gyms, convention, or exhibition centers, have all been put into use [25]. Now, all confirmed cases have received medical treatment in China. China’s strong command system has ensured efficient decisions and synchronized policies in fight COVID-19 [26]. The lockdown of Wuhan city effectively reduced the number of infections in China [27, 28]. The free nucleic acid test, the strict quarantine of all confirmed patients as well as suspected cases and close contacts, and all patients could be admitted to hospitals in Wuhan city with makeshift hospitals built and existing ones expanded, helped to control the infection spread. The Chinese government also mobilized efforts to boost medical supplies and daily necessities to Hubei province. A mechanism was established to organize pairing assistance from other provinces to Hubei’s cities other than Wuhan for the treatment of the infected. Assistance from 19 provinces was rendered to 16 cities in Hubei. On February 11, 2020, the supply of medical protective suits to Hubei exceeded its needs. The public complies with the measures, such as staying at home, maintaining social distancing, wearing a mask, and washing hands frequently, making the prevention and control measures a reality. These positive prevention and control measures have achieved notable outcomes since January 23, 2020.

## Conclusions

In this paper, the difference of spatial transmission characteristics of SARS-CoV-2 in China at the early outbreak stage with prevention and control measures are discussed. Simultaneously, the COVID-19 trend of China at the early time was described from the statistical perspective using a mathematical model. The epidemic modeling given by the GGM model indicates that the early outbreak of COVID-19 exhibits both global polynomial and local exponential growth patterns. The differences between the estimated basic reproduction number also indicate that COVID-19 are sustaining localized outbreaks in some neighboring provinces and the ones with related industry chain and close economic partnership. The consecutive decline of the new confirmed cases indicates that the prevention and control measures taken by the China government have contained the spread of SARS-CoV-2 in a short period.

## Data Availability

The datasets used and/or analyzed during the current study are available from the corresponding author on request.

## Abbreviations

COVID-19: Novel coronavirus disease 2019
SARS-CoV-2: Severe acute respiratory syndrome coronavirus 2
CNHC: The National Health Committee of the People’s Republic of China
GGM: Generalized-growth model
SEIR: Susceptible-Exposed-Infectious-Recovered
SIR: Susceptible-infected-recovered
SARS: Severe acute respiratory syndrome
MERS: Middle East respiratory syndrome
AI: Artificial intelligence
RNA: Ribonucleic acid
